# Drug resistance gene mutations and treatment outcomes in MDR-TB: A prospective study in Eastern China

**DOI:** 10.1371/journal.pntd.0009068

**Published:** 2021-01-20

**Authors:** Qiao Liu, Dandan Yang, Beibei Qiu, Leonardo Martinez, Ye Ji, Huan Song, Zhongqi Li, Jianming Wang

**Affiliations:** 1 Department of Epidemiology, Center for Global Health, School of Public Health, Nanjing Medical University, Nanjing, PR China; 2 Department of Sexually Transmitted Diseases and AIDS, Center for Disease Control and Prevention of Jiangsu Province, Nanjing, PR China; 3 Division of Infectious Diseases and Geographic Medicine, School of Medicine, Stanford University, Stanford, California, United States of America; Faculty of Science, Ain Shams University (ASU), EGYPT

## Abstract

**Background:**

Multidrug-resistant tuberculosis (MDR-TB) poses a serious challenge to TB control. It is of great value to search for drug resistance mutation sites and explore the roles that they play in the diagnosis and prognosis of MDR-TB.

**Methods:**

We consecutively enrolled MDR-TB patients from five cities in Jiangsu Province, China, between January 2013 and December 2014. Drug susceptibility tests of rifampin, isoniazid, ofloxacin, and kanamycin were routinely performed by proportion methods on Lowenstein–Jensen (LJ) medium. Drug resistance-related genes were sequenced, and the consistency of genetic mutations and phenotypic resistance was compared. The association between mutations and treatment outcomes was expressed as odds ratios (ORs) and 95% confidence intervals (CIs).

**Results:**

Among 87 MDR-TB patients, 71 with treatment outcomes were involved in the analysis. The proportion of successful treatment was 50.7% (36/71). The *rpoB* gene exhibited the highest mutation rate (93.0%) followed by *katG* (70.4%), *pncA* (33.8%), *gyrA* (29.6%), *eis* (15.5%), *rrs* (12.7%), *gyrB* (9.9%) and *rpsA* (4.2%). Multivariable analysis demonstrated that patients with *pncA* gene mutations (adjusted OR: 19.69; 95% CI: 2.43–159.33), advanced age (adjusted OR: 13.53; 95% CI: 1.46–124.95), and nonstandard treatment (adjusted OR: 7.72; 95% CI: 1.35–44.35) had a significantly higher risk of poor treatment outcomes.

**Conclusions:**

These results suggest that *Mycobacterium tuberculosis* gene mutations may be related to phenotypic drug susceptibility. The *pncA* gene mutation along with treatment regimen and age are associated with the treatment outcomes of MDR-TB.

## Author summary

Multidrug-resistant tuberculosis (MDR-TB) exacerbates the already serious tuberculosis epidemic, poses a notable threat to global tuberculosis control, and places a considerable burden on developing countries, as treatments for MDR-TB tend to be expensive, of limited efficacy, and toxic. Genotypic determinants of resistance to specific drugs or drug classes offer a rapid and highly specific alternative to phenotypic drug susceptibility testing. Although the relationship between gene mutations and drug resistance has been described previously, the strength of the association of mutations with the treatment outcomes of MDR tuberculosis have not been fully elucidated. The results of our study, which was conducted in a Chinese population, suggest that gene mutations in *Mycobacterium tuberculosis* may be related to phenotypic drug susceptibility. Mutation of the *pncA* gene contributes to a poor prognosis and can be applied to predict the treatment outcomes of MDR-TB.

## Introduction

Tuberculosis (TB) remains a leading cause of death globally, particularly in low- and low-middle-income countries [1]. The increasing incidence of multidrug-resistant TB (MDR-TB), which is defined as resistance to at least isoniazid (INH) and rifampin (RIF), poses a serious challenge to TB control. In a nationwide survey across China, the prevalence of MDR-TB was 5.7% among new cases and 25.6% among previously treated cases [2].

The treatment of drug-resistant TB is usually more complex, toxic, and costly, as well as less effective, than the treatment of other forms of TB [3,4]. Both multidrug resistance and rifampin resistance alone are associated with an inadequate response to first-line treatment [5]. The critical regimens employed to treat MDR-TB include fluoroquinolones (FQs), second-line injectable anti-TB drugs (SLID), and pyrazinamide (PZA) [6,7]. Resistance to these drugs can lead to a prolonged treatment period and may increase the risk of unfavorable outcomes [7].

Gene mutations in *Mycobacterium tuberculosis (M*.*tb)* contribute to phenotypic drug susceptibility. The majority of mutations related to FQ resistance are located in the *gyrA* and *gyrB* genes [8]. Resistance to SLID is associated with gene mutations in rrs and eis, while resistance to PZA is associated with *pncA* and *rpsA* [9,10]. Recent studies have demonstrated that different mutations in *M*.*tb*, even those occurring within the same region, can confer different degrees of phenotypic resistance to anti-TB drugs [11–13]. Therefore, the combination of mutations at multiple sites has a comprehensive effect on drug resistance. We hypothesize that drug-resistant gene mutations can predict the drug-resistant phenotype and the prognosis of MDR-TB.

Although the relationship between gene mutations and drug resistance has been reported previously, the effect of common mutations on the treatment outcomes of MDR-TB has not been elucidated. In this study, we compared the frequency of mutations in common drug resistance-related genes with the *in vitro* drug susceptibility test (DST) results and evaluated their roles in predicting the treatment outcomes of MDR-TB. In this study, we enrolled a group of MDR-TB patients and sequenced the mutation sites of the first- and second-line anti-TB drug-related genes in *M*.*tb*. The objective of this study was to investigate the relationship between gene mutations and the drug-resistant phenotype and determine its role in predicting the prognosis of MDR-TB.

## Methods

### Ethics approval and consent to participate

This study was approved by the Ethics Committee of Nanjing Medical University (approval number: 2019–225). Written informed consent was obtained from all participants. This study was conducted in accordance with the Declaration of Helsinki.

### Study subjects

A prospective cohort study was conducted in five cities in Jiangsu Province, China. Newly registered MDR-TB patients were enrolled from TB designated hospitals, including the Third People's Hospital of Changzhou, the People's Hospital of Taizhou, the Sixth People's Hospital of Nantong, the Infectious Disease Hospital of Xuzhou, and the Fourth People's Hospital of Lianyungang. MDR-TB was identified by regional reference laboratories using the traditional DST. Each patient signed an informed consent form followed by a questionnaire-based survey to gather demographic characteristics and clinical data. TB strains were isolated and transported to the Center for Disease Control and Prevention (CDC) of Jiangsu Province for verification and gene sequencing. Patients were followed for the treatment outcomes. Cure or completion of treatment was defined as treatment success. Death, treatment failure, discontinuation of treatment due to adverse reactions, or loss of follow-up were defined as unfavorable (poor) outcomes. Patients who achieved cured or completed treatment had a follow-up time ranging from 20 months to 26 months.

### Inclusion and exclusion criteria

Patients with MDR-TB detected by DST who could be followed up were eligible for the study. Those who were HIV-positive or had severe complications and were unable to participate in the study were excluded.

### Strain identification and DST

MTB was cultured and identified by p-nitrobenzoic acid (PNB) and thiophene carboxylic acid hydrazine resistance tests. Growth in Lowenstein-Jensen (LJ) medium containing PNB indicates that the bacilli do not belong to the MTB complex. Species other than MTB were excluded from the current analysis. DST was performed using a conventional proportion method on LJ medium according to the guidelines of the WHO. The LJ medium was impregnated with INH, RIF, kanamycin (KM), and ofloxacin (OFX). The concentrations of anti-TB drugs were 0.2 μg/ml for INH, 40 μg/ml for RIF, 30 μg/ml for KM and 2 μg/ml for OFX. The strain was defined as sensitive when the growth rate was < 1% compared to the control. Otherwise, the strain was declared resistant to the specific drug. For internal quality assurance of DST, a standard H37Rv strain was included in each new batch of the LJ medium.

### DNA sequencing of drug resistance-related genes

Mutations in the *rpoB*, *katG*, *inhA*, *pncA*, *rpsA*, *gyrA*, *gyr*B, *rrs*, and *eis* genes were analyzed by PCR amplification and then sequenced using the respective oligonucleotide primers (S1 Table). Primers were designed by referring to previous studies[14–19]. Cycling conditions are shown in S2 Table. PCR products were sent to Sangon Biotech (Shanghai, China) for sequencing and aligned to the H37Rv reference strain (GenBank accession no. NC 000962) using ApE (v2.0.55, http://jorgensen.biology.utah.edu/wayned/ape/).

### Treatment regimens

The standard treatment of MDR-TB contains the intensive and continuation phases, ranging from 24 months to 27 months, depending on the length of the intensive phase [20]. Drugs used to treat MDR-TB included Pyrazinamide (PZA), Ethambutol (EMB), KM, Amikacin (Am), Capreomycin (Cm), OFX, Levofloxacin (Lfx), Moxifloxacin (Mfx), Cycloserine (Cs), Para-aminosalicylic acid (PAS), and Protionamide (Pto). Patients were treated with standardized or individualized regimens based on their treatment histories and DST results.

### Definitions

We defined new cases as patients who had never been treated for TB or had taken anti-TB drugs for less than one month. Previously treated patients were defined as those who had received one month or more of anti-TB drugs in the past. MDR-TB was defined as TB with resistance to at least both INH and RIF. Extensively drug-resistant TB (XDR-TB) was defined as TB with resistance to at least INH, RIF, KM, and OFX. Treatment outcomes were categorized according to the guidelines by the WHO [21]. Standard and individual treatment regimens were designed according to WHO guidelines [22,23]. Nonstandard treatment was defined as any MDR-TB treatment regimen that did not conform with the standard treatment regimen described previously. A "cured" patient was defined as one who had completed treatment according to the program protocol and had no evidence of treatment failure. Three or more consecutive cultures taken at least 30 days apart after the intensive phase should be negative. A "treatment completed" patient was defined as one who had completed treatment according to the program protocol but did not meet the definition of “cured” because of a lack of bacteriological results. The category of "died" comprised any patient who died for any reason during treatment. The "treatment failure" was recorded if the treatment was terminated or needed a permanent regimen change for at least two anti-TB drugs. The "lost to follow-up" category comprised any patient whose treatment was interrupted for two consecutive months or more. The category of "transferred out" comprised any patient transferred to another health facility and was unable to provide feedback on treatment outcomes. For the purpose of the analysis, we combined “cured” and “completed treatment” as "treatment success", whereas other outcomes were grouped as "poor treatment outcomes".

### Sample and data collection

Three sputum samples were collected from all suspected TB patients from regional TB designated hospitals in five cities followed by sputum smear detection and culture. Eligible MDR-TB patients were enrolled from TB designated hospitals and confirmed by a regional reference laboratory using traditional DST. MTB strains were isolated and transported to the Jiangsu CDC for cross-checking and gene sequencing. *M*.*tb* strains were stored for an extended time in a cryopreserved solution composed of tryptone and glycerol at -70°C. Laboratory results, including sputum smear, culture, and DST results, were managed with the Jiangsu Provincial TB Laboratory Data Management System. Epidemiological data were input with EpiData 3.1 software (www.epidata.dk).

### Statistical analysis

We designed a questionnaire to collect demographic characteristics, disease history, treatment history, and behaviors of study subjects. We collected sputum smear, culture, and DST results from each TB reference laboratory. Treatment regimens and outcomes were recorded in the TB management system. After checking the questionnaire data, laboratory data, follow-up data, and sequencing data, a database was formed for analysis. Categorized variables were described using proportions. We used the logistic regression model to estimate the association and expressed it with odds ratios (ORs) and 95% confidence intervals (CIs). All statistical analyses were performed using SPSS 25.0 (IBM, NY, USA).

## Results

### Characteristics of study subjects

**Fig 1:**
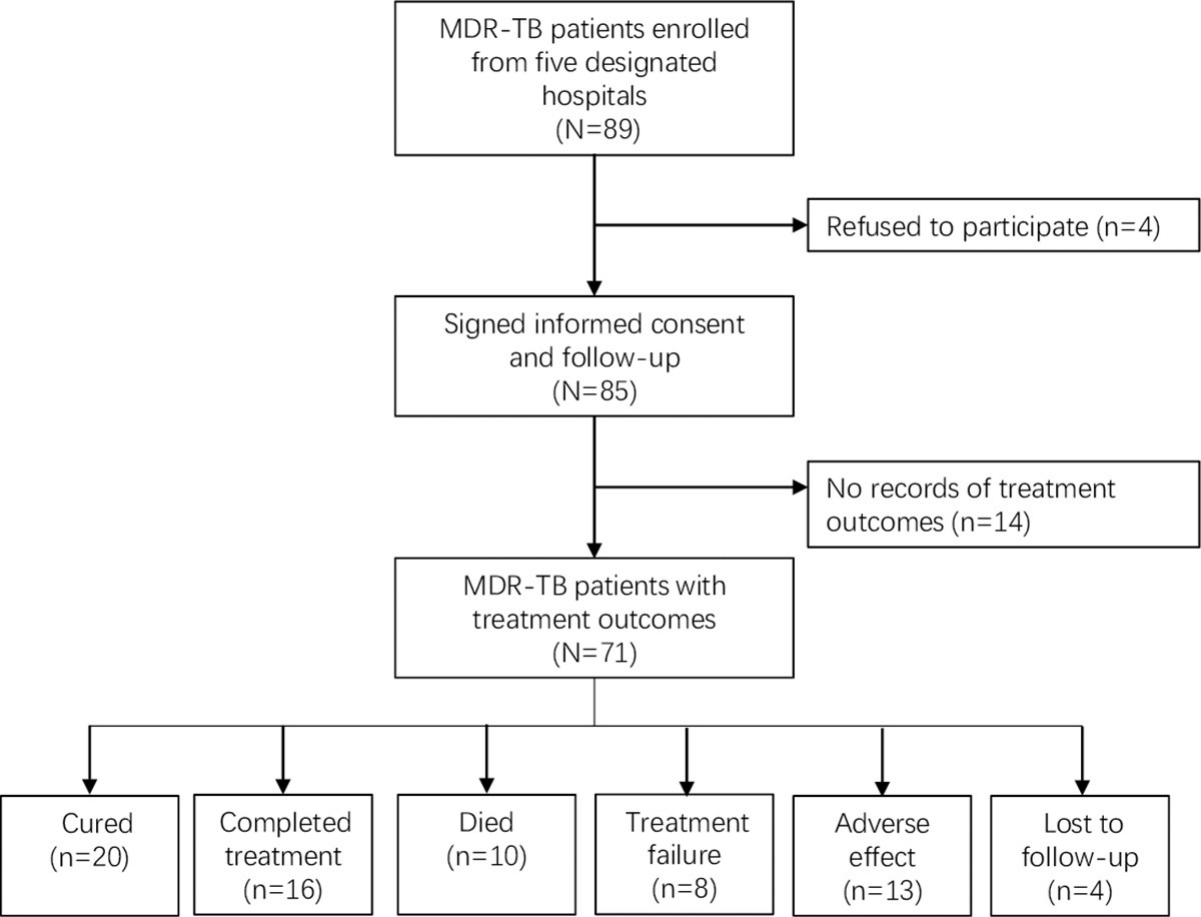
Eligibility and enrollment of study subjects MDR-TB: multidrug-resistant tuberculosis.

A total of 89 MDR-TB patients were recruited during the study period. After excluding the patients who could not meet the inclusion criteria, 71 patients were included in the final analysis (Fig 1). Among these patients, 24 (33.8%) were younger than 44 years, 52 (73.2%) were male, 29 (40.9%) were newly treated, 47 (66.2%) had an alcohol drinking history, 37 (52.1%) had a tobacco smoking history, and 48 (67.6%) were treated with the standard regimen recommended by the WHO. The proportion of successful treatment was 50.7%. Of the 35 patients with poor outcomes, 10 died, 8 had treatment failure, 13 had an adverse effect, and 4 were lost to follow-up (Table 1).

**Table 1:** Demographic characteristics of 71 multidrug-resistant tuberculosis patients.

Variable	Total, N (%)	Treatment success, N (%)	Poor outcomes, N (%)
Sex			
Male	52 (73.2)	27 (75.0)	25(71.4)
Female	19 (26.8)	9 (25.0)	10 (28.6)
Age (years)			
<44	24 (33.8)	15 (41.7)	9 (25.7)
44–54	25 (35.2)	13 (36.1)	12 (34.3)
>54	22 (31.0)	8 (22.2)	14 (40.0)
Weight (kg)			
<55	28 (39.4)	10 (27.8)	18 (51.4)
55–60	23 (32.4)	16 (44.4)	7 (20.0)
>60	20 (28.2)	10 (27.8)	10 (28.6)
History of contact			
Yes	6 (8.4)	4 (11.1)	2 (5.7)
No	65 (91.6)	32 (88.9)	33 (94.3)
Smoking			
Yes	37 (52.1)	20 (55.6)	17 (48.6)
No	34 (47.9)	16 (44.4)	18 (51.4)
Drinking			
Yes	47 (66.2)	21 (58.3)	26 (74.3)
No	12 (16.9)	7 (19.4)	5 (14.3)
Missing	12 (16.9)	8 (22.2)	4 (11.4)
Treatment history			
New cases	29 (40.9)	19 (52.8)	10 (28.6)
Previously treated	42 (59.2)	17 (47.2)	25(71.4)
Standard treatment			
Yes	48 (67.6)	30 (83.3)	18 (51.4)
No	23 (32.4)	6 (16.7)	17 (48.6)
Sputum smear grade			
<3+	40 (56.3)	17 (47.2)	23 (65.7)
≥3+	31 (43.7)	19 (52.8)	12 (34.3)

### Phenotypic drug resistance identified by DST

There were 20 (28.2%) strains resistant to OFX and 8 (11.3%) strains resistant to KM. A total of 51 (71.8%) isolates were only resistant to both INH and RIF, which was defined as simple MDR-TB. Seventeen (23.9%) MDR-TB isolates that were additionally resistant to either OFX or KM were defined as pre-XDR-TB, and 3 (4.2%) MDR-TB isolates that were additionally resistant to both OFX and KM were defined as XDR-TB.

### DNA sequencing

The most prevalent mutations were located in *rpoB*531 (41/71; 62.1%) for RIF, *katG*315 (47/71; 66.2%) and C (–15) T in *inhA* (8/71; 11.3%) for INH; *gyrA*94 (9/71; 12.7%) for OFX; *rrs*1401 (4/71; 5.6%) and -22 in *eis* (10/71; 14.1%) for KM (Table 2). The proportion of mutations related to resistance to RIF, INH, PZA, OFX and KM was 93.0% (66/71), 77.5% (55/71), 38.0% (27/71), 38.0% (27/71), and 23.9% (17/71), respectively.

**Table 2:** Genotypic and phenotypic characteristics of resistance to anti-tuberculosis drugs.

Drug	Gene	Locus	Nucleotide/codon change	Nucleotide/amino acid change	No of isolates
RIF	*rpoB*	459	CTG-CGG	LeT-Arg	1
		463	CAG-CAC	Gln-His	1
		511	CTG-CCG	LeT-Pro	5
		513	CAA-CCA	Gln-Pro	1
		513	CAA-CTA	Gln-LeT	1
		515	ATG-CTG	Met-LeT	1
		516	GAC-TAC	Asp-Tyr	2
		516	GAC-GTC	Asp-Val	5
		522	TCG-TTG	Ser-Let	2
		526	CAC-TAC	His-Tyr	2
		526	CAC-AAC	His-Asn	3
		526	CAC-GAC	His-Asp	2
		526	CAC-CGC	His-Arg	2
		526	CAC-CTC	His-LeT	3
		526	CAC-CAG	His-Gln	1
		531	TCG-TTG	Ser-LeT	41
		533	CTG-CCC	LeT-Pro	2
INH	*katG*	241	CCC-CGC	Pro-Arg	1
		289	GAG-GGG	GlT-Gly	1
		291	GCT-GTT	Ala-Val	1
		315	AGC-ACC	Ser-Thr	47
	*inhA*	-15	ACG-ATG	Thr-Met	8
PZA	*pncA*	41	GGT-GGA	Gly-Gly	1
		44	GCA-GCC	Ala-Ala	2
		53	CCG-CCA	Pro-Pro	1
		58	TCT-CCT	Ser-Pro	2
		62	AAT-AAG	Asn-Lys	1
		63	GAT-GAC	Asp-Asp	1
		64	AT_-ATC	_-Ile	1
		114	CCA-CCG	Pro-Pro	1
		114	CCA-CCC	Pro-Pro	1
		124	TAC-GAC	Tyr-Asp	1
		127	CGC-CGT	Arg-Arg	1
		155	CGA-CGT	Arg-Arg	1
		164	CCA-CCG	Pro-Pro	1
		187	AGT-AGC	Ser-Ser	2
		192	CGA-GGA	Arg-Gly	1
		195	TCA-TCG	Ser-Ser	1
		198	GCA-GCG	Ala-Ala	1
		201	CAC-C_C	His-	1
	*rpsA*	10	_CC-CCC	_-Pro	1
		15	AAT-AGT	Asn-Ser	1
		119	AGA-ACA	Arg-Thr	1
		234	_CC-CCC	_-Pro	1
		240	_CC-CCC	_-Pro	1
OFX	*gyrA*	45	CTC-CTG	LeT-LeT	1
		60	TTC-CTC	LeT-LeT	2
		90	GCG-GTG	Ala-Val	7
		93	ACG-ACC	Thr-Thr	1
		93	ACG-ACT	Thr-Thr	3
		94	ACA-GCA	Thr-Ala	6
		94	ACA-CCA	Thr-Pro	2
		94	ACA-ATA	Thr-Ile	1
	*gyrB*	509	AAC-ACC	Asn-Thr	2
		514	GCG-GTG	Ala-Val	1
		522	GGG-AGG	Gly-Arg	4
KM	*rrs*	1205	_-A	_-Met	1
		1227	_-T	_-LeT	1
		1401	A-G	Thr-Ala	4
		1449	A-G	Arg-Gly	1
		1449	T-C	Ser-Pro	1
		1601	T-A	Ser-Arg	1
	*eis*	-57	_-G	_-Ser	1
		-54	C-G	Asp-GlT	2
		-22	A-_	Asn-_	10
		2	A-_	Lys-_	1

RIF: rifampin; INH: isoniazid; PZA: promethazine; FQ: fluoroquinolone; KM; kanamycin.

DNA sequencing identified 17 mutations in the *rpoB* gene from 66 (93.0%) isolates, with the most common one being at codon 531 (Ser→Leu) (41/71, 57.8%). Four types of mutations were identified in the *katG* gene from 50 (70.4%) isolates, with the most common one being at codon 315 (47/71, 66.2%). Double substitutions in *katG* and *inhA* were observed in 3 isolates. Among strains resistant to PZA, 24 (88.9%) carried mutations in *pncA*, and 3 (11.1%) carried mutations in *rpsA*. Among strains resistant to OFX, 21 (77.8%) carried mutations in *gyrA*, 7 (25.9%) carried mutations in *gyrB*, and 1 (3.7%) had mutations in *gyrA* and *gyrB*. Among strains resistant to KM, 9 (52.9%) had mutations in the *rrs* gene, and 14 (82.4%) had mutations in the *eis* gene (Table 2). Compared with DST, DNA sequencing exhibited a relatively good performance (S3 Table).

### Factors related to treatment outcomes

As shown in Table 3, previous treatment history (OR: 2.79, 95% CI: 1.05–7.47, *P* = 0.04) and receiving a nonstandard treatment regimen (OR: 4.72, 95% CI: 1.55–14.17, *P* = 0.01) contributed to a poor treatment outcome. We also observed that *pncA* gene mutation (OR: 5.29, 95% CI: 1.76–15.89, *P* <0.01), *pncA* gene mutation (OR: 5.29, 95% CI: 1.76–15.89, *P* <0.01), and *gyrA* gene mutation (OR: 3.75, 95% CI: 1.25–11.30, *P* = 0.02) were related to a poor outcome (Table 4). We further explored the role of high-frequency mutation sites in the prognosis of MDR-TB but without significant findings (S4 Table).

**Table 3:** Univariable logistic regression analysis of demographic factors associated with treatment outcomes.

Variable	Total, N (%)	Treatment success, N (%)	Poor outcomes, N (%)	cOR (95% CI)	*P*
Sex					
Male	52 (73.2)	27 (75.0)	25(71.4)	Reference	
Female	19 (26.8)	9 (25.0)	10 (28.3)	1.20 (0.42–3.44)	0.73
Age (years)					
<44	24 (33.8)	15 (41.7)	9 (25.7)	Reference	
44–54	25 (35.2)	13 (36.1)	12 (34.3)	1.54 (0.49–4.81)	0.46
>54	22 (31.0)	8 (22.2)	14 (40.0)	2.92 (0.88–9.67)	0.08
Weight (kg)					
<55	28 (39.4)	10 (27.8)	18 (51.4)	Reference	
55–60	23 (32.4)	16 (44.4)	7 (20.0)	0.24 (0.08–0.79)	0.02
>60	20 (28.2)	10 (27.8)	10 (28.6)	0.56 (0.17–1.79)	0.32
History of contact					
No	65 (91.6)	32 (88.9)	33 (94.3)	Reference	
Yes	6 (8.4)	4 (11.1)	2 (5.7)	0.49 (0.08–2.83)	0.42
Smoking					
No	34 (47.9)	16 (44.4)	18 (51.4)	Reference	
Yes	37 (52.1)	20 (55.6)	17 (48.6)	0.76 (0.30–1.92)	0.56
Treatment history					
New cases	29 (40.9)	19 (52.8)	10 (28.6)	Reference	
Previously treated	42 (59.2)	17 (47.2)	25(71.4)	2.79 (1.05–7.47)	0.04
Standard treatment					
Yes	48 (67.6)	30 (83.3)	18 (51.4)	Reference	
No	23 (32.4)	6 (16.7)	17 (48.6)	4.72 (1.55–14.17)	0.01
Baseline sputum smear test					
<3+	40 (56.3)	17 (47.2)	23 (65.7)	Reference	
≥3+	31 (43.7)	19 (52.8)	12 (34.3)	0.47 (0.18–1.22)	0.12

cOR: crude odds ratio, CI: confidence interval, TB: tuberculosis

**Table 4:** Univariable logistic regression analysis of drug resistance gene mutations associated with treatment outcomes.

Variable	Total, N (%)	Treatment success, N (%)	Poor outcomes, N (%)	cOR (95% CI)	*P*
*rpoB* gene mutation					
No	5 (7.0)	2 (5.6)	3 (8.6)	Reference	
Yes	66 (93.0)	34 (94.4)	32 (91.4)	0.63 (0.01–4.00)	0.62
*katG* gene mutation					
No	21 (29.6)	11 (30.6)	10 (28.6)	Reference	
Yes	50 (70.4)	25 (69.4)	25 (71.4)	1.10 (0.40–3.05)	0.86
*inhA* gene mutation					
No	63 (88.7)	33 (91.7)	30 (85.7)	Reference	
Yes	8 (11.3)	3 (8.3)	5 (14.3)	1.83 (0.40–8.34)	0.43
*katG* and *inhA* genes mutation					
No	16 (22.5)	9 (25.0)	7 (20.0)	Reference	
Yes	55 (77.5)	27 (75.0)	28 (80.0)	1.33 (0.44–4.01)	0.62
*pncA* gene mutation					
No	47 (66.2)	30 (83.3)	17 (48.6)	Reference	
Yes	24 (33.8)	6 (16.7)	18 (51.4)	5.29 (1.76–15.89)	<0.01
*rpsA* gene mutation					
No	68 (95.8)	34 (94.4)	34 (97.1)	Reference	
Yes	3 (4.2)	2 (5.6)	1 (2.9)	0.50 (0.04–5.78)	0.58
*pncA* or *rpsA* gene mutation					
No	44 (70.4)	28 (77.8)	16 (45.7)	Reference	
Yes	27 (29.6)	8 (22.2)	19 (54.3)	4.16 (1.49–11.64)	<0.01
*gyrA* gene mutation					
No	50 (67.6)	30 (83.3)	20 (57.1)	Reference	
Yes	21 (32.4)	6 (16.7)	15 (42.9)	3.75 (1.25–11.30)	0.02
*gyrB* gene mutation					
No	64 (90.1)	32 (88.9)	32 (91.4)	Reference	
Yes	7 (9.9)	4 (11.1)	3 (8.6)	0.75 (0.16–3.62)	0.72
*gyrA* and *gyrB* gene mutation					
No	44 (62.0)	26 (72.2)	18 (51.4)	Reference	
Yes	27 (38.0)	10 (27.8)	17 (48.6)	2.46 (0.92–6.58)	0.09
*rrs* gene mutation					
No	62 (87.3)	30 (83.3)	32 (91.4)	Reference	
Yes	9 (12.7)	6 (16.7)	3 (8.6)	0.47 (0.11–2.04)	0.31
*eis* gene mutation					
No	60 (84.5)	31 (86.1)	29 (82.9)	Reference	
Yes	11 (15.5)	5 (13.9)	6 (17.1)	1.28 (0.35–4.66)	0.71
*rrs* and *eis* genes mutation					
No	54 (76.1)	26 (72.2)	28 (80.0)	Reference	
Yes	17 (23.9)	10 (27.8)	7 (20.0)	0.65 (0.22–1.96)	0.44

cOR: crude odds ratio; CI: confidence interval

After adjusting for potential confounders based on a multivariate logistic regression model, we observed that patients with advanced age (≥ 54 years) (adjusted OR: 13.53, 95% CI: 1.46–124.95, *P* = 0.02), receiving nonstandard treatment (adjusted OR: 7.72, 95% CI: 1.35–44.35, *P* = 0.02) and having mutations in the *pncA* gene (adjusted OR: 19.69, 95% CI: 2.43–159.33, *P* < 0.01) had an increased risk of poor treatment outcomes (Table 5).

**Table 5:** Multivariable logistic regression analysis of drug resistance gene mutations and treatment outcomes.

Variable	Total, N (%)	Treatment success, N (%)	Poor outcomes, N (%)	aOR (95% CI)	*P*
Sex					
Male	52 (73.2)	27 (75.0)	25(71.4)	Reference	
Female	19 (26.8)	9 (25.0)	10 (28.3)	2.14 (0.28–16.35)	0.46
Age (years)					
<44	24 (33.8)	15 (41.7)	9 (25.7)	Reference	
44–54	25 (35.2)	13 (36.1)	12 (34.3)	7.02 (0.87–56.62)	0.67
>54	22 (31.0)	8 (22.2)	14 (40.0)	13.53 (1.46–124.95)	**0.02**
Weight (kg)					
<55	28 (39.4)	10 (27.8)	18 (51.4)	Reference	
55–60	23 (32.4)	16 (44.4)	7 (20.0)	0.18 (0.02–1.40)	0.10
>60	20 (28.2)	10 (27.8)	10 (28.6)	0.40 (0.06–2.79)	0.36
History of contact					
No	65 (91.6)	32 (88.9)	33 (94.3)	Reference	
Yes	6 (8.4)	4 (11.1)	2 (5.7)	5.24 (0.30–91.89)	0.26
Smoking					
No	34 (47.9)	16 (44.4)	18 (51.4)	Reference	
Yes	37 (52.1)	20 (55.6)	17 (48.6)	0.66 (0.11–3.86)	0.65
Treatment history					
New cases	29 (40.9)	19 (52.8)	10 (28.6)	Reference	
Previously treated	42 (59.2)	17 (47.2)	25(71.4)	0.75 (0.10–5.46)	0.77
Standard treatment					
Yes	48 (67.6)	30 (83.3)	18 (51.4)	Reference	
No	23 (32.4)	6 (16.7)	17 (48.6)	7.72 (1.35–44.35)	**0.02**
Sputum smear grade					
<3+	40 (56.3)	17 (47.2)	23 (65.7)	Reference	
≥3+	31 (43.7)	19 (52.8)	12 (34.3)	0.34 (0.06–1.78)	0.20
*rpoB* gene mutation					
No	5 (7.0)	2 (5.6)	3 (8.6)	Reference	
Yes	66 (93.0)	34 (94.4)	32 (91.4)	0.82 (0.01–53.17)	0.93
*katG* gene mutation					
o	21 (29.6)	11 (30.6)	10 (28.6)	Reference	
Yes	50 (70.4)	25 (69.4)	25 (71.4)	0.39 (0.04–4.26)	0.44
*inhA* gene mutation					
No	63 (88.7)	33 (91.7)	30 (85.7)	Reference	
Yes	8 (11.3)	3 (8.3)	5 (14.3)	0.67 (0.07–6.60)	0.73
*pncA* gene mutation					
No	47 (66.2)	30 (83.3)	17 (48.6)	Reference	
Yes	24 (33.8)	6 (16.7)	18 (51.4)	19.69 (2.43–159.33)	**<0.01**
*rpsA* gene mutation					
No	68 (95.8)	34 (94.4)	34 (97.1)	Reference	
Yes	3 (4.2)	2 (5.6)	1 (2.9)	0.96 (0.02–40.16)	0.98
*gyrA* gene mutation					
No	50 (67.6)	30 (83.3)	20 (57.1)	Reference	
Yes	21 (32.4)	6 (16.7)	15 (42.9)	3.50 (0.512–23.94)	0.20
*gyrB* gene mutation					
No	64 (90.1)	32 (88.9)	32 (91.4)	Reference	
Yes	7 (9.9)	4 (11.1)	3 (8.6)	0.49 (0.02–15.52)	0.69
*rrs* gene mutation					
No	62 (87.3)	30 (83.3)	32 (91.4)	Reference	
Yes	9 (12.7)	6 (16.7)	3 (8.6)	0.12 (0.01–1.78)	0.12
*eis* gene mutation					
No	60 (84.5)	31 (86.1)	29 (82.9)	Reference	
Yes	11 (15.5)	5 (13.9)	6 (17.1)	0.93 (0.13–6.84)	0.94

aOR: adjusted odds ratio; CI: confidence interval

## Discussion

MDR-TB poses a significant challenge to TB control. It takes several weeks for traditional DST to obtain results; therefore, it is of great value to search biomarkers for rapid detection and prediction of MDR-TB. A better understanding of the association between genotypic resistance to anti-TB drugs and treatment outcomes could facilitate the selection of effective drugs. We found that patients with advanced age, receiving nonstandard treatment, or infected with strains having mutations in PZA-related genes were at increased risk of poor treatment outcomes. PZA is a sterilizing drug with similar effectiveness as RIF. The *pncA* gene encodes pyrazinamidase, which converts PZA into its active form, pyrazinoic acid. Mutations in the *pncA* gene are the dominant mechanism of drug resistance to PZA, which has been found in > 90% of PZA-resistant isolates [24,25].

In our study, half of the MDR-TB patients with PZA resistance were also at a high risk of resistance to FQs and SLID, indicating the role of the *pncA* mutation as a biomarker for MDR-TB. Similar findings have been observed in Chongqing, China, where PZA resistance was frequently accompanied by resistance to OFX, AM, KM, and CM [26], and *pncA* mutations were found in more than half of MDR-TB cases [27]. A study in South Africa showed that 69% of MDR-TB had *pncA* mutations, and 96% of XDR-TB had *pncA* mutations [28]. In a study in Shanghai, the mutation rate of the *pncA* gene was 37.8% in patients with MDR-TB [9]. PZA is used in susceptible TB treatment when used in combination with RIF, INH, and EMB and is a critical companion drug in new MDR-TB trials [29]. At present, there are few studies on the phenotype and genotype of PZA resistance in association with MDR-TB.

Correlation of drug resistance with a defect in a specific “drug-resistant” gene has been observed for INH, RIF, EMB, and OFX [30]. However, PZA appears to have multiple cellular targets, resulting in a variable correlation between PZA resistance and mutations in the *pncA* gene. Approximately 72–97% of PZA-resistant strains had *pncA* gene mutations. These mutated loci are numerous and scattered in the *pncA* gene [31]. In addition to *pncA*, other genes, such as *rpsA*, *panD*, and *hadC*, have also been reported to be related to PZA resistance, but with inconsistency. Alexander believes that *rpsA* does not have a necessary ability to detect PZA resistance [32], while Wanliang notes that *rpsA* mediates relatively low-frequency mutations but can still detect PZA resistance [33]. A study in southern China found that a high-frequency mutation occurred at the 3' end of *rpsA* without *pncA* gene mutation, suggesting the value of *rpsA* in detecting PZA resistance [26]. The role of *rpsA*, *panD*, and *hadC* mutations with wild-type *pncA* genes in PZA resistance warrants further investigation [30].

FQs are considered the most critical component of MDR-TB treatment regimens, including OFX, Mfx, and Lfx. The DNA gyrase of *M*.*tb*, encoded by *gyrA* and *gyrB*, is well established as the quinolone target. The detection of mutations in the *gyrA* and *gyrB* genes has been demonstrated to be a promising technology for the rapid diagnosis of FQ resistance. In this study, we also observed that *gyrA* gene mutation was a risk factor for poor prognosis of MDR-TB, and the high-frequency mutation site was *gyrA*94. Treatment outcomes are primarily affected by the drug resistance level mediated by *gyrA* mutations. A study conducted by Harvard University found that high-level drug resistance caused by *gyrA* gene mutations was strongly associated with poor treatment outcomes. In contrast, the moderate-level resistance mediated by *gyrA* gene mutation did not show a significant statistical effect [34]. A multinational cohort study also suggested the role of high-level resistance-related *gyrA* mutations increased the risk of death for MDR/XDR-TB patients [35]. High doses of Mfx added to the MDR-TB treatment regimen can prevent patients from developing XDR-TB, indicating that low concentrations of FQ resistance caused by mutations in the *gyrA* locus can be treated with high doses of Mfx [36].

Although this study is the first to explore the genetic polymorphisms of dominant second-line anti-TB drug resistance genes in the treatment outcomes of MDR-TB in China, before generalizing the findings, we should not overlook its limitations. First, the sample size was relatively small, and these results must be confirmed in more extensive studies. Second, we did not perform MIC analyses to correlate gene mutations with the conferred resistance levels specifically. Third, we only conducted DST for INH, RIF, KM, and OFX and could not analyze the consistency between gene mutation and drug resistance of other anti-TB drugs. However, such biases were probably nondifferential with a tendency to diminish the strength of associations, rather than inflating them.

In conclusion, gene mutations in *M*.*tb* are related to phenotypic drug susceptibility. The *pncA* gene mutation, together with treatment regimen and age, can be applied to predict the prognosis of MDR-TB. Findings from this study suggest that drug-resistant gene testing should be conducted for MDR-TB patients to adjust the treatment regimens in a timely manner and improve the prognosis of patients. In China, new anti-TB drugs, such as bedaquiline and delamanid, are now being applied to treat MDR-TB. However, DST is not routinely performed to identify the drug resistance of these novel drugs. If molecular biology techniques can be widely utilized in clinical practice, they will help guide drug use and improve prognosis.

## Supporting information

S1 Table DNA sequencing primers for anti-tuberculosis drug resistance genes. (DOCX)

S2 Table Cycling conditions of anti-tuberculosis drug resistance genes. (DOCX)

S3 Table Performance of DNA sequencing compared to phenotypic DST. (DOCX)

S4 Table Univariate logistic analysis of high-frequency mutation sites and treatment outcomes. (DOCX)

## References

[1] Uplekar M, Weil D, Lonnroth K, Jaramillo E, Lienhardt C, Dias HM, et al WHO's new end TB strategy. Lancet. 2015;385(9979):1799–801. Epub 2015/03/31. 10.1016/S0140-6736(15)60570-0 . 25814376

[2] Zhao Y, Xu S, Wang L, Chin DP, Wang S, Jiang G, et al National survey of drug-resistant tuberculosis in China. N Engl J Med. 2012;366(23):2161–70. Epub 2012/06/08. 10.1056/NEJMoa1108789 . 22670902

[3] Wingfield T, Boccia D, Tovar M, Gavino A, Zevallos K, Montoya R, et al Defining catastrophic costs and comparing their importance for adverse tuberculosis outcome with multi-drug resistance: a prospective cohort study, Peru. PLoS Med. 2014;11(7):e1001675 Epub 2014/07/16. 10.1371/journal.pmed.1001675 25025331 PMC4098993

[4] Ding P, Li X, Jia Z, Lu Z. Multidrug-resistant tuberculosis (MDR-TB) disease burden in China: a systematic review and spatio-temporal analysis. BMC Infect Dis. 2017;17(1):57 Epub 2017/01/12. 10.1186/s12879-016-2151-5 28073344 PMC5223590

[5] Menzies D, Benedetti A, Paydar A, Royce S, Pai M, Burman W, et al Standardized treatment of active tuberculosis in patients with previous treatment and/or with mono-resistance to isoniazid: a systematic review and meta-analysis. PLoS Medicine. 2009;6(9). 10.1371/journal.pmed.1000150 20101802 PMC2736403

[6] Hwang SS, Kim HR, Kim HJ, Kim MJ, Lee SM, Yoo CG, et al Impact of resistance to first-line and injectable drugs on treatment outcomes in MDR-TB. Eur Respir J. 2009;33(3):581–5. Epub 2009/03/03. 10.1183/09031936.00099608 . 19251799

[7] Falzon D, Gandhi N, Migliori GB, Sotgiu G, Cox HS, Holtz TH, et al Resistance to fluoroquinolones and second-line injectable drugs: impact on multidrug-resistant TB outcomes. Eur Respir J. 2013;42(1):156–68. Epub 2012/10/27. 10.1183/09031936.00134712 23100499 PMC4487776

[8] Kocagoz T, Hackbarth CJ, Unsal I, Rosenberg EY, Nikaido H, Chambers HF. Gyrase mutations in laboratory-selected, fluoroquinolone-resistant mutants of Mycobacterium tuberculosis H37Ra. Antimicrob Agents Chemother. 1996;40(8):1768–74. Epub 1996/08/01. 10.1128/AAC.40.8.1768 8843279 PMC163415

[9] Zheng X, Ning Z, Drobniewski F, Yang J, Li Q, Zhang Z, et al pncA mutations are associated with slower sputum conversion during standard treatment of multidrug-resistant tuberculosis. Int J Antimicrob Agents. 2017;49(2):183–8. Epub 2016/12/26. 10.1016/j.ijantimicag.2016.10.012 . 28012685

[10] Rigouts L, Coeck N, Gumusboga M, de Rijk WB, Aung KJ, Hossain MA, et al Specific gyrA gene mutations predict poor treatment outcome in MDR-TB. J Antimicrob Chemother. 2016;71(2):314–23. Epub 2015/11/26. 10.1093/jac/dkv360 26604243 PMC4710215

[11] Kambli P, Ajbani K, Nikam C, Sadani M, Shetty A, Udwadia Z, et al Correlating rrs and eis promoter mutations in clinical isolates of Mycobacterium tuberculosis with phenotypic susceptibility levels to the second-line injectables. International journal of mycobacteriology. 2016;5(1):1–6. 10.1016/j.ijmyco.2015.09.001 26927983 PMC4863938

[12] Kambli P, Ajbani K, Sadani M, Nikam C, Shetty A, Udwadia Z, et al Defining multidrug-resistant tuberculosis: correlating GenoType MTBDRplus assay results with minimum inhibitory concentrations. Diagn Microbiol Infect Dis. 2015;82(1):49–53. Epub 2015/03/10. 10.1016/j.diagmicrobio.2015.01.009 25749461 PMC4414878

[13] Kambli P, Ajbani K, Sadani M, Nikam C, Shetty A, Udwadia Z, et al Correlating Minimum Inhibitory Concentrations of ofloxacin and moxifloxacin with gyrA mutations using the genotype MTBDRsl assay. Tuberculosis (Edinb). 2015;95(2):137–41. Epub 2014/12/20. 10.1016/j.tube.2014.11.003 25522842 PMC4361297

[14] Yoshida S, Suzuki K, Iwamoto T, Tsuyuguchi K, Tomita M, Okada M, et al Comparison of rifabutin susceptibility and rpoB mutations in multi-drug-resistant Mycobacterium tuberculosis strains by DNA sequencing and the line probe assay. J Infect Chemother. 2010;16(5):360–3. Epub 2010/04/01. 10.1007/s10156-010-0057-5 . 20354890

[15] Zhao LL, Sun Q, Zeng CY, Chen Y, Zhao B, Liu HC, et al Molecular characterisation of extensively drug-resistant Mycobacterium tuberculosis isolates in China. Int J Antimicrob Agents. 2015;45(2):137–43. Epub 2014/12/04. 10.1016/j.ijantimicag.2014.09.018 . 25465521

[16] Mokrousov I, Otten T, Filipenko M, Vyazovaya A, Chrapov E, Limeschenko E, et al Detection of isoniazid-resistant Mycobacterium tuberculosis strains by a multiplex allele-specific PCR assay targeting katG codon 315 variation. J Clin Microbiol. 2002;40(7):2509–12. Epub 2002/06/29. 10.1128/jcm.40.7.2509-2512.2002 12089271 PMC120554

[17] Akhmetova A, Kozhamkulov U, Bismilda V, Chingissova L, Abildaev T, Dymova M, et al Mutations in the pncA and rpsA genes among 77 Mycobacterium tuberculosis isolates in Kazakhstan. Int J Tuberc Lung Dis. 2015;19(2):179–84. Epub 2015/01/13. 10.5588/ijtld.14.0305 . 25574916

[18] Chen J, Chen Z, Li Y, Xia W, Chen X, Chen T, et al Characterization of gyrA and gyrB mutations and fluoroquinolone resistance in Mycobacterium tuberculosis clinical isolates from Hubei Province, China. Braz J Infect Dis. 2012;16(2):136–41. Epub 2012/05/04. 10.1016/s1413-8670(12)70294-5 . 22552454

[19] Chakravorty S, Lee JS, Cho EJ, Roh SS, Smith LE, Lee J, et al Genotypic susceptibility testing of Mycobacterium tuberculosis isolates for amikacin and kanamycin resistance by use of a rapid sloppy molecular beacon-based assay identifies more cases of low-level drug resistance than phenotypic Lowenstein-Jensen testing. J Clin Microbiol. 2015;53(1):43–51. Epub 2014/10/24. 10.1128/JCM.02059-14 25339395 PMC4290905

[20] Velayutham B, Nair D, Kannan T, Padmapriyadarsini C, Sachdeva K. S, Bency J, et al Factors associated with sputum culture conversion in multidrug-resistant pulmonary tuberculosis. Int J Tuberc Lung Dis 2016 p. 1671–6. 10.5588/ijtld.16.0096 27931345

[21] WHO. Definitions and reporting framework for tuberculosis– 2013 revision: updated December 2014 and January 2020 Geneva: WHO; 2013 [cited 2020 October 22]. Available from: https://apps.who.int/iris/handle/10665/79199.

[22] WHO. Guidelines for the programmatic management of drug-resistant tuberculosis. Geneva: World Health Organization; 2011. 23844450

[23] WHO. Treatment of tuberculosis: guidelines. 4th ed Geneva: World Health Organization; 2010. 23741786

[24] Chang KC, Yew WW, Zhang Y. Pyrazinamide susceptibility testing in Mycobacterium tuberculosis: a systematic review with meta-analyses. Antimicrob Agents Chemother. 2011;55(10):4499–505. Epub 2011/07/20. 10.1128/AAC.00630-11 21768515 PMC3186960

[25] Scorpio A, Lindholm-Levy P, Heifets L, Gilman R, Siddiqi S, Cynamon M, et al Characterization of pncA mutations in pyrazinamide-resistant Mycobacterium tuberculosis. Antimicrob Agents Chemother. 1997;41(3):540–3. Epub 1997/03/01. 10.1128/AAC.41.3.540 9055989 PMC163747

[26] Tan Y, Hu Z, Zhang T, Cai X, Kuang H, Liu Y, et al Role of pncA and rpsA gene sequencing in detection of pyrazinamide resistance in Mycobacterium tuberculosis isolates from southern China. J Clin Microbiol. 2014;52(1):291–7. Epub 2013/10/18. 10.1128/JCM.01903-13 24131688 PMC3911430

[27] Pang Y, Zhu D, Zheng H, Shen J, Hu Y, Liu J, et al Prevalence and molecular characterization of pyrazinamide resistance among multidrug-resistant Mycobacterium tuberculosis isolates from Southern China. BMC Infect Dis. 2017;17(1):711 Epub 2017/11/08. 10.1186/s12879-017-2761-6 29110640 PMC5674869

[28] Jacobson KR, Theron D, Victor TC, Streicher EM, Warren RM, Murray MB. Treatment outcomes of isoniazid-resistant tuberculosis patients, Western Cape Province, South Africa. Clin Infect Dis. 2011;53(4):369–72. Epub 2011/08/04. 10.1093/cid/cir406 21810750 PMC3202325

[29] Juma SP, Maro A, Pholwat S, Mpagama SG, Gratz J, Liyoyo A, et al Underestimated pyrazinamide resistance may compromise outcomes of pyrazinamide containing regimens for treatment of drug susceptible and multi-drug-resistant tuberculosis in Tanzania. BMC Infect Dis. 2019;19(1):129 Epub 2019/02/09. 10.1186/s12879-019-3757-1 30732572 PMC6367741

[30] Njire M, Tan Y, Mugweru J, Wang C, Guo J, Yew W, et al Pyrazinamide resistance in Mycobacterium tuberculosis: Review and update. Adv Med Sci. 2016;61(1):63–71. Epub 2015/11/02. 10.1016/j.advms.2015.09.007 . 26521205

[31] Li D, Hu Y, Werngren J, Mansjo M, Zheng X, Drobniewski F, et al Multicenter Study of the Emergence and Genetic Characteristics of Pyrazinamide-Resistant Tuberculosis in China. Antimicrob Agents Chemother. 2016;60(9):5159–66. Epub 2016/06/15. 10.1128/AAC.02687-15 27297481 PMC4997820

[32] Alexander DC, Ma JH, Guthrie JL, Blair J, Chedore P, Jamieson FB. Gene sequencing for routine verification of pyrazinamide resistance in Mycobacterium tuberculosis: a role for pncA but not rpsA. J Clin Microbiol. 2012;50(11):3726–8. Epub 2012/08/17. 10.1128/JCM.00620-12 22895038 PMC3486241

[33] Shi W, Zhang X, Jiang X, Yuan H, Lee JS, Barry CE 3rd, et al Pyrazinamide inhibits trans-translation in Mycobacterium tuberculosis. Science. 2011;333(6049):1630–2. Epub 2011/08/13. 10.1126/science.1208813 21835980 PMC3502614

[34] Tsj O, Rjj K, Jh C, M W, Fm VZ. Myocardial blood flow and myocardial flow reserve values in (13)N-ammonia myocardial perfusion PET/CT using a time-efficient protocol in patients without coronary artery disease. Eur J Hybrid Imaging. 2018;2(1):11 Epub 2018/06/29. 10.1186/s41824-018-0029-z 29951642 PMC5994394

[35] Georghiou SB, Seifert M, Catanzaro DG, Garfein RS, Rodwell TC. Increased Tuberculosis Patient Mortality Associated with Mycobacterium tuberculosis Mutations Conferring Resistance to Second-Line Antituberculous Drugs. J Clin Microbiol. 2017;55(6):1928–37. Epub 2017/04/14. 10.1128/JCM.00152-17 28404672 PMC5442550

[36] Jo KW, Lee SD, Kim WS, Kim DS, Shim TS. Treatment outcomes and moxifloxacin susceptibility in ofloxacin-resistant multidrug-resistant tuberculosis. Int J Tuberc Lung Dis. 2014;18(1):39–43. Epub 2013/12/25. 10.5588/ijtld.13.0307 . 24365550

