# Supplementary material for: Drug resistance gene mutations and treatment outcomes in MDR-TB: A prospective study in Eastern China
Source: PLoS Negl Trop Dis. 2021 Jan 20;15(1):e0009068. doi: 10.1371/journal.pntd.0009068 (PMC7850501; doi:10.1371/journal.pntd.0009068)
Supplement: S1 Table — (DOCX) [file pntd.0009068.s001.docx]

S1 Table. DNA sequencing primers for anti-tuberculosis drug resistance genes

| **Gene** | **Primer** | **Sequence (5’→3’)** | **Product size (bp)** |
| --- | --- | --- | --- |
| rpoB | rpoB-F | CGACCACTTCGGCAACCG | 342bp |
|  | rpoB-R | TCGATCGGGCACATCCGG |  |
| katG | katG-F | AATCGATGGGCTTCAAGACG | 550bp |
|  | katG-R | CTCGTAGCCGTACAGGATCTCG |  |
| inhA | inhA-F | TGCCCAGAAAGGGATCCGTCATG | 455bp |
|  | inhA-R | ATGAGGAATGCGTCCGCGGA |  |
| pncA | pncA-F | GCCGCCAACAGTTCATCC | 760bp |
|  | pncA-R | GATTTGTCGCTCACTACATCACC |  |
| rpsA | rpsA-F | ATCTGTCCCTACGACCCAAC | 742bp |
|  | rpsA-R | ACCGTCCACACCGCCGAGA |  |
| gyrA | gryA-F | ATCGAGCAGGAGATGCAG | 553bp |
|  | gryA-R | CGTCGTAGTTAGGGATGAAA |  |
| gyrB | gryB-F | GTTTGAAGCCAACCCCACC | 470bp |
|  | gryB-R | TGAACCGGAACAACAACGT |  |
| rrs | rrs-F | GTCAACTCGGAGGAAGGTGG | 516bp |
|  | rrs-R | GTCCGAGTGTTGCCTCAGG |  |
| eis | eis-F | CACAGGGTCACAGTCACAGAATC | 98bp |
|  | eis-R | GCATCGCGTGATCCTTTGCCAGAC |  |
