# Supplementary material for: Drug resistance gene mutations and treatment outcomes in MDR-TB: A prospective study in Eastern China
Source: PLoS Negl Trop Dis. 2021 Jan 20;15(1):e0009068. doi: 10.1371/journal.pntd.0009068 (PMC7850501; doi:10.1371/journal.pntd.0009068)
Supplement: S2 Table — (DOCX) [file pntd.0009068.s002.docx]

S2 Table. Cycling conditions of anti-tuberculosis drug resistance genes

| Genes | Cycling conditions |
| --- | --- |
| rpoB, katG | 35 cycles at 94°C for 1 min, annealing for 1 min at 60°C, then elongation at 72°C for 1 min |
| inhA, rrs, eis | 25 cycles at 94°C for 1 min; annealing for 1 min at 60°C, then elongation at 72°C for 1 min. |
| pncA, rpsA | 38 cycles at 95°C for 10 seconds; annealing for 10 seconds at 64°C, then elongation at 72°C for 20 seconds |
| gyrA, gyrB | 25 cycles at 96°C for 10 seconds; annealing for 5 seconds at 50°C, then elongation at 60°C for 4 min. |
