# Supplementary material for: Drug resistance gene mutations and treatment outcomes in MDR-TB: A prospective study in Eastern China
Source: PLoS Negl Trop Dis. 2021 Jan 20;15(1):e0009068. doi: 10.1371/journal.pntd.0009068 (PMC7850501; doi:10.1371/journal.pntd.0009068)
Supplement: S3 Table — (DOCX) [file pntd.0009068.s003.docx]

S3 Table. Performance of DNA sequencing compared to phenotypic DST

| Molecular methods | | Phenotypic DST result | | | | | |
| --- | --- | --- | --- | --- | --- | --- | --- |
|  |  | Resistant | Susceptible | Sensitivity % (95% CI) | Specificity % (95% CI) | PPV % (95% CI) | NPV % (95% CI) |
| RFP | Resistant | 66 | 0 | 92.96 (84.3-97.7) | / | 100 | / |
|  | Susceptible | 5 | 0 |  |  |  |  |
| INH | Resistant | 55 | 0 | 77.46(66.0-86.5) | / | 100 | / |
|  | Susceptible | 16 | 0 |  |  |  |  |
| MDR | Resistant | 55 | 0 | 77.46(66.0-86.5) | **/** | 100 | / |
|  | Susceptible | 16 | 0 |  |  |  |  |
| OFX | Resistant | 18 | 9 | 90.0(68.3-98.8) | 82.4 (69.1-91.6) | 66.7 (46.0-83.5) | 95.5(84.5-99.4) |
|  | Susceptible | 2 | 42 |  |  |  |  |
| KM | Resistant | 6 | 11 | 75.0 (34.9-96.8) | 82.5 (70.9-90.9) | 35.3 (14.2-61.7) | 96.3 (87.3-99.5) |
|  | Susceptible | 2 | 52 |  |  |  |  |

Abbreviations: RFP, rifampicin; INH, isoniazid; MDR, multidrug resistant; OFX, ofloxacin; KM, kanamycin; CI, confidence interval; DST, drug susceptibility testing; PPV, positive predictive value; NPV, negative predictive value.
