# Supplementary material for: Drug resistance gene mutations and treatment outcomes in MDR-TB: A prospective study in Eastern China
Source: PLoS Negl Trop Dis. 2021 Jan 20;15(1):e0009068. doi: 10.1371/journal.pntd.0009068 (PMC7850501; doi:10.1371/journal.pntd.0009068)
Supplement: S4 Table — (DOCX) [file pntd.0009068.s004.docx]

S4 Table. Univariate logistic analysis of high frequency mutation sites and treatment outcomes.

| **Mutations** | **Successful treatment N (%)** | **Undesirable treatment outcome N (%)** | **cOR (95% CI)** | ***P*** |
| --- | --- | --- | --- | --- |
| rpoB531 | 22 (61.1) | 19(54.3) | 0.76 (0.29-1.94) | 0.56 |
| katG315 | 24(66.7) | 23(65.7) | 0.96 (0.36-3.56) | 0.93 |
| inhA-15 | 3(8.3) | 5(14.3) | 1.83 (0.40-8.34) | 0.43 |
| gyrA90 | 1(2.8) | 6(17.1) | 7.24 (0.82-63.64) | 0.07 |
| gyrA94 | 2(5.6) | 7(20.0) | 4.25 (0.82-22.11) | 0.09 |
| rrs1401 | 2(5.6) | 2(5.7) | 1.03 (0.14-7.75) | 0.98 |
| eis-22 | 5(13.9) | 5(14.3) | 1.03 (0.27-3.94) | 0.96 |

cOR=crude Odds Ratio, CI=Confidence Interval
