# Supplementary material for: Drug resistance gene mutations and treatment outcomes in MDR-TB: A prospective study in Eastern China
Source: PLoS Negl Trop Dis. 2021 Jan 20;15(1):e0009068. doi: 10.1371/journal.pntd.0009068 (PMC7850501; doi:10.1371/journal.pntd.0009068)
Supplement: Attachment — Submitted filename: Reply20201025.docx [file pntd.0009068.s005.docx]

**Liu Qiao *et al.* “Drug Resistance Gene Mutations and Treatment Outcomes of MDR-TB: A Prospective Study in Eastern China” (your reference no. PNTD-D-20-01499), Point-by-point response**

25 October 2020

Dear editor and reviewers,

We are pleased to submit a revised version of our manuscript, entitled “Drug Resistance Gene Mutations and Treatment Outcomes of MDR-TB: A Prospective Study in Eastern China” (PNTD-D-20-01499). We thank you and the external reviewers for the rapid turn-around of our report, the careful examination, helpful comments, and the invitation to resubmit a revised manuscript.

We have carefully and fully addressed the editorial comments and comments from Reviewers 1 and 2. Please find our point-by-point response, clearly indicating the changes made to the manuscript below.

With this manuscript, we would like to confirm that the material has not and will not be offered elsewhere for possible publication while under consideration in your journal. We also confirm that all the contributing authors have reviewed this revision and concur with the revised submission made by the corresponding author.

Please contact the corresponding author (Dr. Jianming Wang) via the above contact details if any further information is needed. We trust that our findings will be of interest to the readership of *PLOS Neglected Tropical Diseases*.

Sincerely,

Jianming Wang

On behalf of all authors:

Qiao Liu, Dandan Yang, Beibei Qiu, Leonardo Martinez, Ye Ji, Huan Song, Zhongqi Li, Jianming Wang

**Response to Editor**

*Comment #1:*

A letter containing a detailed list of your responses to the review comments and a description of the changes you have made in the manuscript. Please note while forming your response, if your article is accepted, you may have the opportunity to make the peer review history publicly available. The record will include editor decision letters (with reviews) and your responses to reviewer comments. If eligible, we will contact you to opt in or out.

**Response:**

We thank the editor and the two reviewers for the constructive comments to our manuscript. Below please find a response letter including line-by-line responses to each of the reviewers” comments.

*Comment #2:*

Two versions of the revised manuscript: one with either highlights or tracked changes denoting where the text has been changed; the other a clean version (uploaded as the manuscript file)

**Response:**

As requested, we have provided two versions of the manuscript, one with changes marked in “Track Changes” and the other as a clean version with no changes marked.

**Reviewer 1:**

*Comment #1:*

Please describe sample collection fully, also how data was collected including the data collection software.

**Response:**

We wish to thank the reviewer for the thoughtful review of our manuscript and suggestions to improve this manuscript. We agree and have now added several sentences to the Methods section, which can be seen below.

**“Sample and data collection**

Three sputum samples were collected from all suspected TB patients from regional TB designated hospitals in five cities followed by sputum smear detection and culture. Eligible MDR-TB patients were enrolled from TB designated hospitals and confirmed by a regional reference laboratory using traditional DST. MTB strains were isolated and transported to the Jiangsu CDC for cross-checking and gene sequencing. *M.tb* strains were stored for an extended time in a cryopreserved solution composed of tryptone and glycerol at -70 ℃. Laboratory results, including sputum smear, culture, and DST results, were managed with the Jiangsu Provincial TB Laboratory Data Management System. Epidemiological data were input with EpiData 3.1 software (www.epidata.dk).”

*Comment #2:*

The objectives of the study are clear and the study design is appropriate. The study population is clearly described and appropriate for the hypothesis. The sample size is small and the authors acknowledged that.

**Response:**

We thank the reviewer for these positive comments and review of our manuscript.

*Comment #3:*

There are concerns regarding data handling. No description on how samples were collected, stored and how data was collected and stored

**Response:**

We agree with the reviewer and have now added several sentences in the Methods section describing the sample collection process.

**“Sample and data collection**

Three sputum samples were collected from all suspected TB patients from regional TB designated hospitals in five cities followed by sputum smear detection and culture. Eligible MDR-TB patients were enrolled from TB designated hospitals and confirmed by a regional reference laboratory using traditional DST. MTB strains were isolated and transported to the Jiangsu CDC for cross-checking and gene sequencing. *M.tb* strains were stored for an extended time in a cryopreserved solution composed of tryptone and glycerol at -70 ℃. Laboratory results, including sputum smear, culture, and DST results, were managed with the Jiangsu Provincial TB Laboratory Data Management System. Epidemiological data were input with EpiData 3.1 software (www.epidata.dk).”

*Comment #4:*

The analysis plan was not described in full. The results are clear and well presented and the figures are clear.

**Response:**

We used questionnaires to collect epidemiological information, including patient’s general demographic characteristics, disease history, and life habits of MDR-TB patients. We received sputum smear results, sputum culture, and traditional drug susceptibility test results from each tuberculosis reference laboratory. We then followed-up MDR-TB patients in the TB patient management system. As part of this follow-up, we collected the treatment regimen and treatment outcomes of each patient. PCR products were sent to Sangon Biotech (Shanghai) Co., Ltd. for gene sequencing. The analytical plan included organizing the data into categorical and continuous variables. We used EpiData 3.1 software to input data through the double entry for consistency. Categorized variables were described using proportions. We used logistic regression models to estimate the association between tuberculosis treatment outcomes and gene mutations. Measures of association were expressed with an odds ratio (OR) and 95% confidence intervals (CI). All statistical analysis was conducted using SPSS software 25.0. We have now added text to the Methods section on the analytical plan. Specifically, we have now included the following text:

“**Statistical analysis**

We designed a questionnaire to collect demographic characteristics, disease history, treatment history, and behaviors of study subjects. We collected sputum smear, culture, and DST results from each TB reference laboratory. Treatment regimens and outcomes were recorded in the TB management system. After checking the questionnaire data, laboratory data, follow-up data, and sequencing data, a database was formed for analysis. Categorized variables were described using proportions. We used the logistic regression model to estimate the association and expressed it with odds ratios (ORs) and 95% confidence intervals (CIs). All statistical analyses were performed using SPSS 25.0 (IBM, NY, USA).”

*Comment #5:*

It is not easy to judge the limitations because the methods were not described explicitly, including what sample was collected and how it was collected.

**Response:**

We agree with the reviewer’s comments and have now added several sentences in the Methods section to describe the sample collection:

**“Sample and data collection**

Three sputum samples were collected from all suspected TB patients from regional TB designated hospitals in five cities followed by sputum smear detection and culture. Eligible MDR-TB patients were enrolled from TB designated hospitals and confirmed by a regional reference laboratory using traditional DST. MTB strains were isolated and transported to the Jiangsu CDC for cross-checking and gene sequencing. *M.tb* strains were stored for an extended time in a cryopreserved solution composed of tryptone and glycerol at -70 ℃. Laboratory results, including sputum smear, culture, and DST results, were managed with the Jiangsu Provincial TB Laboratory Data Management System. Epidemiological data were input with EpiData 3.1 software (www.epidata.dk).*”*

Further information and changes can be seen in response to Comment #3.

*Comment #6:*

The authors also do not fully address how the data will advance understanding of the topic and the public health relevance of the study

**Response:**

In China, PZA is commonly administered in combination with SLDs for the treatment of MDR-TB. Until now, phenotypic sensitivity testing of pyrazinamide has not been a routine test item in China, and MDR-TB patients know little about pyrazinamide drug resistance during daily treatment. The pncA gene mutation related to PZA drug resistance has been reported previously; however, the association between treatment outcomes and gene mutations among MDR-TB patients is not well elucidated. Our study suggests that genotype sensitivity should be included in tuberculosis clinical diagnosis and may be of use to improve treatment regimens and predict unfavorable treatment outcomes among MDR-TB patients. Due to the concern of the reviewer, we have now added several sentences to the Discussion on this topic. Specifically, we now state it in the revised manuscript as the following:

“Findings from this study suggest that drug-resistant gene testing should be conducted for MDR-TB patients to adjust the treatment regimens in a timely manner and improve the prognosis of patients. In China, new anti-TB drugs, such as bedaquiline and delamanid, are now being applied to treat MDR-TB. However, DST is not routinely performed to identify the drug resistance of these novel drugs. If molecular biology techniques can be widely utilized in clinical practice, they will help guide drug use and improve prognosis.”

*Comment #7:*

The citations in the paper are not done well and cannot be linked to the references. Authors should please familiarise themselves with their referencing style including doing citations.

**Response:**

We have made substantial changes to the citations in the paper be linked to the references.

*Comment #8:*

The study is important in adding to the body of knowledge on drug-resistant TB considering that the studies available have low numbers of participants. Minor revision is needed.

**Response:**

We thank the reviewer for this comment and their overall review. We believe, as a result, the manuscript has substantially improved.

**Reviewer 2.**

*Comment #1:*

The objectives of the study are clearly stated; however, I did not see the author’s hypothesis or maybe it is not well articulated.

**Response:**

We wish to thank the reviewer for the thoughtful review of our manuscript and suggestions to improve this manuscript. We now include several sentences in the Introduction section describing the hypothesis of the manuscript. Specifically, we have now included the following text:

“We hypothesize that drug-resistant gene mutations can predict the drug-resistant phenotype and the prognosis of MDR-TB.

Although the relationship between gene mutations and drug resistance has been reported previously, the effect of common mutations on the treatment outcomes of MDR-TB has not been elucidated. In this study, we compared the frequency of mutations in common drug resistance-related genes with the *in vitro* drug susceptibility test (DST) results and evaluated their roles in predicting the treatment outcomes of MDR-TB. In this study, we enrolled a group of MDR-TB patients and sequenced the mutation sites of the first- and second-line anti-TB drug-related genes in *M.tb*. The objective of this study was to investigate the relationship between gene mutations and the drug-resistant phenotype and determine its role in predicting the prognosis of MDR-TB.”

*Comment #2:*

It is also not very clear how long the patients were followed; were they followed up until they completed their treatment?

**Response:**

A total of 89 MDR-TB patients were recruited during the study period. After excluding the patients who could not meet the inclusion criteria, 71 patients were included in the final analysis. Of 35 patients with poor treatment outcomes, 10 died, 8 had treatment failure, 13 had an adverse effect, and 4 were lost to follow-up. The remaining 36 patients who achieved cured or completed treatment were followed up until the completion of treatment, with follow-up time ranging from 20 months to 26 months. To clarify, we have now added several sentences to the Methods section detailing the follow-up duration of patients in the study.

“Patients were followed for the treatment outcomes. Cure or completion of treatment was defined as treatment success. Death, treatment failure, discontinuation of treatment due to adverse reactions, or loss of follow-up were defined as unfavorable (poor) outcomes. Patients who achieved cured or completed treatment had a follow-up time ranging from 20 months to 26 months.”

*Comment #3:*

The authors need to clarify if patients with drug resistance other than MDR were excluded from the study i.e. what is the exclusion criteria?

**Response:**

Only MDR-TB patients resistant to both RIF and INH were included in our study. We have added inclusion criteria and exclusion criteria in the Method section as the following:

“**Inclusion and exclusion criteria**

Patients with MDR-TB detected by DST who could be followed up were eligible for the study. Those who were HIV-positive or had severe complications and were unable to participate in the study were excluded.”

*Comment #4:*

The authors need to state the relevance of their research to public health or rather clearly state how their data can be used to improve patient management/management of MDR TB cases.

**Response:**

We agree with the reviewer and have now added text to the Discussion section.

“Findings from this study suggest that drug-resistant gene testing should be conducted for MDR-TB patients to adjust the treatment regimens in a timely manner and improve the prognosis of patients. In China, new anti-TB drugs, such as bedaquiline and delamanid, are now being applied to treat MDR-TB. However, DST is not routinely performed to identify the drug resistance of these novel drugs. If molecular biology techniques can be widely utilized in clinical practice, they will help guide drug use and improve prognosis.”

*Comment #5:*

It would also be important for the authors to address their findings in the context of newer TB drugs for the treatment of MDR TB, such as bedaquiline and delamanid.

**Response:**

In China, new anti-TB drugs such as bedaquiline and delamanid are now being used in MDR-TB clinical treatment. Similarly, currently, there is no DST method to detect the drug resistance of these drugs. If the detection of drug-resistant related gene mutation sites can be incorporated into clinical treatment, it may be better to guide the treatment of MDR-TB patients. We have discussed it in the revised manuscript.

“In conclusion, gene mutations in *M.tb* are related to phenotypic drug susceptibility. The *pncA* gene mutation, together with treatment regimen and age, can be applied to predict the prognosis of MDR-TB. Findings from this study suggest that drug-resistant gene testing should be conducted for MDR-TB patients to adjust the treatment regimens in a timely manner and improve the prognosis of patients. In China, new anti-TB drugs, such as bedaquiline and delamanid, are now being applied to treat MDR-TB. However, DST is not routinely performed to identify the drug resistance of these novel drugs. If molecular biology techniques can be widely utilized in clinical practice, they will help guide drug use and improve prognosis.”

*Comment #6:*

What do their findings mean for patients who are being treated with newer TB drugs?

**Response:**

This is an important point as the use of newer TB drugs is changing in China. In China, new anti-TB drugs such as bedaquiline and delamanid are now being used in MDR-TB clinical treatment. Similarly, DST is not currently used to detect the drug resistance of these novel drugs. If the detection of drug-resistant related gene mutation sites can be incorporated into clinical treatment, it may assist in guiding the use of these drugs to improve the treatment of MDR-TB patients. Due to the concern of the reviewer, we have now added text to the Discussion on this topic.

“In China, new anti-TB drugs, such as bedaquiline and delamanid, are now being applied to treat MDR-TB. However, DST is not routinely performed to identify the drug resistance of these novel drugs. If molecular biology techniques can be widely utilized in clinical practice, they will help guide drug use and improve prognosis.”

*Comment #7:*

Authors need to clarify a few points that were raised before the paper is accepted for publication.

**Response:**

We have responded to each reviewer’s comments in a step-by-step process and have made further changes according to reviewer’s suggestions.
