# Supplementary material for: Drug resistance gene mutations and treatment outcomes in MDR-TB: A prospective study in Eastern China
Source: PLoS Negl Trop Dis. 2021 Jan 20;15(1):e0009068. doi: 10.1371/journal.pntd.0009068 (PMC7850501; doi:10.1371/journal.pntd.0009068)
Supplement: Attachment — Submitted filename: Response_20201205.docx [file pntd.0009068.s006.docx]

**Liu Qiao *et al.* “Drug Resistance Gene Mutations and Treatment Outcomes of MDR-TB: A Prospective Study in Eastern China” (your reference no. PNTD-D-20-01499), Point-by-point response**

1 December 2020

Dear Dr. Abdallah M. Samy,

We are pleased to submit a revised version of our manuscript, entitled “Drug Resistance Gene Mutations and Treatment Outcomes of MDR-TB: A Prospective Study in Eastern China” (your reference no. PNTD-D-20-01499). We thank you and the external reviewers for the rapid turn-around of our report, the careful examination, helpful comments, and the invitation to resubmit a revised manuscript.

We have carefully and fully addressed the editorial comments and comments from Reviewers 2. Please find our point-by-point response, clearly indicating the changes made to the manuscript below.

With this manuscript, we the co-authors would like to confirm that the material has not and will not be offered elsewhere for possible publication while under consideration in your journal. We also confirm that all the contributing authors have reviewed this revision and concur with the revised submission made by the corresponding author.

Please contact the corresponding author (Dr. Jianming Wang) via the above contact details if any further information is needed. We trust that our findings will be of interest to the readership of *PLOS Neglected Tropical Diseases*.

Sincerely,

Jianming Wang

On behalf of all authors:

Qiao Liu, Dandan Yang, Beibei Qiu, Leonardo Martinez, Ye Ji, Huan Song, Zhongqi Li, Jianming Wang

**___________________________________________________________**

**Response to Editor.**

*Comment #1:*

A letter containing a detailed list of your responses to the review comments and a description of the changes you have made in the manuscript. Please note while forming your response, if your article is accepted, you may have the opportunity to make the peer review history publicly available. The record will include editor decision letters (with reviews) and your responses to reviewer comments. If eligible, we will contact you to opt in or out.

**Response:** We thank the editor and reviewers for the constructive comments to our manuscript. Below please find a response letter including line-by-line responses to each of the reviewers’ comments.

*Comment #2:*

Two versions of the revised manuscript: one with either highlights or tracked changes denoting where the text has been changed; the other a clean version (uploaded as the manuscript file)

**Response:** As requested, we have provided two versions of the manuscript; one with changes clearly marked in ‘Track Changes’ and the other as a clean version with no changes marked.

**External Reviewer 2:**

*Comment #1:*

The objectives of the study are clearly stated, and the study design is appropriate to address the objectives. The sample size is rather small however the authors have acknowledged this limitation. Authors have used the correct statistical tools which support their conclusions. This study was carried out without any ethical concerns, appropriate ethical clearance was obtained.

**Response:** We thank the Reviewer for these positive comments and review of our manuscript.

*Comment #2:*

The analysis presented matches the analysis plan and the results are clearly presented. However, the authors should clearly explain what they mean by nonstandard treatment (Line 260). The tables and figures are clear and easy to understand and interpret. Overall the results are well presented.

**Response:** We thank the Reviewer for the thoughtful review of our manuscript and helpful suggestions to improve this manuscript. Treatment of MDR-TB patients was done according to WHO Guidelines [1]. This included at least four second-line antituberculosis drugs likely to be effective as well as pyrazinamide during the intensive phase of treatment. The standard treatment regimen is 6 PZA Am (Km, Cm) Lfx (Ofx) Cs (PAS) Pto (EMB)/18 PZA Lfx (Ofx) Cs (PAS) Pto (EMB). In addition, individually designed regimens are given to some MDR-TB patients based on the patient’s history of past drug use and on DST of isoniazid, rifampicin, the second-line injectable agents and a fluoroquinolone. Individual regimens were also given according to WHO guidelines [2]. In addition to the two cases mentioned above, other multidrug resistant treatment regiments were defined as nonstandard treatment.

We have now added text to the manuscript due to the Reviewer’s comment.

Specifically, we now state, “standard and individual treatment regimens were designed according to WHO guidelines [1, 2]. Nonstandard treatment was defined as any MDR-TB treatment regimen that did not conform with the standard treatment regimen described previously.”

*Comment #3:*

Conclusion made are supported by the data that is presented, and authors have clearly outlined the limitations of the study. The authors have discussed clearly how the data presented can help improve our understanding of TB drug resistance and the importance of checking for drug resistance mutations. the public health relevance of the study has been addressed; authors have stated how drug resistant gene testing can be used to improve patient treatment outcomes.

**Response:** We thank the Reviewer for these positive comments and review of our manuscript.

*Comment #4:*

Overall the data that is presented in this manuscript is very important and relevant and helps us understand the impact of genetic mutations on treatment outcomes. This type of work is very important in this era of increased TB drug resistance and also offers a potential alternative to the usual phenotypic drug susceptibility testing.

**Response:** We thank the Reviewer for the thoughtful review of our manuscript and helpful suggestions to improve this manuscript.

References

1.	WHO. Guidelines for the programmatic management of drug-resistant tuberculosis. Geneva: World Health Organization; 2011.

2.	WHO. Treatment of tuberculosis: guidelines. 4th ed. Geneva: World Health Organization; 2010.
